# Estimating the Burden of Japanese Encephalitis Virus and Other Encephalitides in Countries of the Mekong Region

**DOI:** 10.1371/journal.pntd.0002533

**Published:** 2014-01-30

**Authors:** Arnaud Tarantola, Flavie Goutard, Paul Newton, Xavier de Lamballerie, Olivier Lortholary, Julien Cappelle, Philippe Buchy

**Affiliations:** 1 Institut Pasteur du Cambodge, Phnom Penh, Cambodia; 2 Centre de coopération internationale en recherche agronomique pour le développement (CIRAD), Département ES, Unité AGIRs, Montpellier, France; 3 Lao-Oxford-Mahosot Hospital-Wellcome Trust Research Unit, Microbiology Laboratory, Mahosot Hospital, Vientiane, Lao PDR and Centre for Tropical Medicine, Nuffield Department of Medicine, Churchill Hospital, University of Oxford, Oxford, United Kingdom; 4 Aix Marseille University, IRD French Institute of Research for Development, EHESP French School of Public Health, UMR_D 190 “Emergence des Pathologies Virales”, Marseille, France; 5 Université René Descartes, Hôpital Necker-Enfants malades, Centre d'Infectiologie Necker Pasteur, IHU Imagine, Labex IBEID, Paris, France; University of Queensland, Australia

## Abstract

Diverse aetiologies of viral and bacterial encephalitis are widely recognized as significant yet neglected public health issues in the Mekong region. A robust analysis of the corresponding health burden is lacking. We retrieved 75 articles on encephalitis in the region published in English or in French from 1965 through 2011. Review of available data demonstrated that they are sparse and often derived from hospital-based studies with significant recruitment bias. Almost half (35 of 75) of articles were on Japanese encephalitis virus (JEV) alone or associated with dengue. In the Western Pacific region the WHO reported 30,000–50,000 annual JEV cases (15,000 deaths) between 1966 and 1996 and 4,633 cases (200 deaths) in 2008, a decline likely related to the introduction of JEV vaccination in China, Vietnam, or Thailand since the 1980s. Data on dengue, scrub typhus and rabies encephalitis, among other aetiologies, are also reviewed and discussed.

Countries of the Mekong region are undergoing profound demographic, economic and ecological change. As the epidemiological aspects of Japanese encephalitis (JE) are transformed by vaccination in some countries, highly integrated expert collaborative research and objective data are needed to identify and prioritize the human health, animal health and economic burden due to JE and other pathogens associated with encephalitides.

## Introduction

Encephalitides are considered a serious public health issue in countries of the Mekong region (here defined as Cambodia, Lao PDR [Laos], Vietnam, Thailand, and Yunnan of PR China). This part of the world is undergoing profound transformations due to remarkable increases in population, tourism, energy consumption, road networks, vehicle density, economic development and deforestation [1]–[3]. In these times of competing priorities and financial constraints for health authorities, can the burden of encephalitis in the Mekong region be estimated?

Various aspects need to be considered when estimating disease burden: collective versus individual burden, the cost of preventive versus curative measures, morbidity and mortality versus financial burden, acute versus chronic disease, human versus animal disease, etc. With the exception of some data on Japanese encephalitis virus, very few prevalence, incidence, cost-ascertainment or clinical follow-up studies seem available on most identified encephalitides circulating in countries of the Mekong region. We reviewed published Medline-referenced resources on encephalitis and meningoencephalitis and used these to complete an overview of the epidemiological situation in this dynamic region of the world.

## Methods

In January 2012, we reviewed articles published in English or French from 1965 to January 1, 2012 and listed in the Medline database. The terms “Southeastern Asia/epidemiology,” “Encephalitis,” “Arboviral encephalitis,” “Encephalitis, viruses,” and “Anti-N-Methyl-D-Aspartate Receptor Encephalitis” were used alone and in combination.

A case of acute encephalitis syndrome was defined as any person presenting a fever with neurological signs (altered mental status AND/OR motor deficit AND/OR sensory deficit AND/OR seizures of new onset, excluding simple febrile seizures) of sudden onset (fewer than seven days). Meningoencephalitis cases present the abovementioned signs plus meningism (nuchal rigidity).

References on human and animal health were taken into consideration. These were consulted and extracted using the Institut Pasteur's distance bibliographical resources or HINARI (http://hinari-gw.who.int). Bibliographical references of consulted articles were checked for pertinence and also reviewed, as needed. Additional queries were made using Google for specific references freely available on the Internet and resources identified while the manuscript was in preparation were also reviewed.

## Results

The MeSH search query found 58,753 references for “Southeastern Asia/epidemiology,” 38,160 references for “Encephalitis” and 140 articles for “Southeastern Asia/epidemiology” and “Encephalitis.” Of these, references were found using “Southeastern Asia/epidemiology” and “Arboviral encephalitis” (N = 66) and “Encephalitis, viruses” (N = 35). Seven articles were excluded as they were in a language other than English or French, and an additional three described neurocysticercosis, botanical repellents, and mosquito species. A further 55 articles were excluded as their title suggested they dealt with outbreaks or disease in countries outside of the Mekong region: Indonesia, Malaysia, Philippines, or Singapore. Of the 75 references examined, 35 dealt with Japanese encephalitis virus, three with dengue, three with both and seven with Nipah virus infection. The 27 remaining references dealt with various encephalitides or were not detailed.

### Japanese encephalitis virus surveillance and estimated burden data

**Japanese encephalitis virus** (JEV) is the most important cause of acute encephalitis in Eastern/Southern Asia. Based on a data review for 1966–1996, the World Health Organization (WHO) estimated that there were 68,000 (15,000 deaths) annually [4]. Based on 1994 population figures and highly varying annual incidence rates documented during research projects, Tsai estimated that in 1994 there were approximately 175,000 cases, over 43,750 deaths, and 78,750 surviving children with disability [5]. Seroprevalence studies reviewed by WHO found almost universal infection by early adulthood in rural endemic areas [4].

There is a high degree of uncertainty regarding the present situation. Only 4,633 cases (200 deaths) were reported to WHO in 2008 from the Western Pacific region [6], due to some high-endemicity countries having introduced JEV vaccination [7], [8] but certainly also to clinical misdiagnosis [9], underdiagnosis, and/or underreporting. There are very few geographical locations in the Mekong countries with JEV epidemiological data with vast swathes of encephalitis terra incognita [10]. Authors have tried to ascertain the size of the at-risk population and the estimated annual number of JEV cases using modelling techniques [5], [11]–[13]. These modelling techniques far underestimate the observed number of cases in a country like Cambodia, where vaccination is not available countrywide and where the surface used for cultivating rice paddy—harbouring competent vectors—has increased by 39% between 2004 and 2011 [14]. Recently, Campbell et al. [11] revisited and complemented Tsai's method, concluding that 67,897 JEV cases occurred per year worldwide. For Cambodia, however, these estimates are based only on published sentinel surveillance data [15], which probably provides an incomplete picture and will lead to severe underestimation. Using Campbell's method, we arrive at an estimated 8,200 cases of symptomatic infection annually for the Mekong region and 563 for Cambodia alone (not shown). In 2012, the number of meningoencephalitis patients documented by the Kantha Bopha Foundation hospitals alone in Phnom Penh and Siem Reap were 2,090 and 1,521, respectively (2013 phone call with D. Laurent, personal communication). The Institut Pasteur du Cambodge (IPC) tested samples from 317 of these patients as part of a research study, finding JEV IgM in patients' cerebrospinal fluid (CSF) and/or serum in 51 (16.1%). Extrapolated to the 3,611 meningoencephalitis cases, this conservative estimate yields a total number of 581 JEV cases at Kantha Bopha hospitals alone. Meningoencephalitis cases are managed throughout Cambodia, whether in public sector or third-tier Foundation hospitals. Another project at Angkor Hospital for Children (Siem Reap) found 15 (15%) JEV positivity among a series of 100 meningoencephalitis cases (blood and/or CSF, 24 July 2013 email exchanges with V. Kumar, personal communication).

An additional 47 (14.8%) children were positive at IPC both for JEV and Dengue virus (DENV) IgM, for which the causative pathogen could be either flavivirus (15 July 2013 email exchanges with Philippe Buchy, personal communication). This illustrates the additional difficulty of diagnosing JEV encephalitis when CSF may contain undetectable amounts of pathogen or genetic material, when patients arrive late in the course of the disease at hospitals with diagnostic capability, when CSF samples are not routinely taken in countries such as Cambodia, and when attempting diagnosis in countries where other cross-reactive flaviviruses such as dengue are endemoepidemic.

### Clinical impact of JEV

The available JEV data are predominantly derived from hospital-based studies and are therefore likely to underestimate community incidence as most JEV infections are asymptomatic and only an estimated 1 in 250–500 infections result in clinical signs [16], [17]. Those signs vary greatly, ranging from a flu-like or dengue-like febrile syndrome—which is frequent in children in the Mekong region—to severe encephalitis, with a case-fatality rate (CFR) estimated at 5–40% (up to 60% for severe forms). Neurological sequelae occur in 30–50% of survivors and are often severe [18]–[22], although it seems that a majority of patients recovered after three to five years in one large study in India [23]. It has been estimated [24] that 265,778–1,859,170 disease-adjusted life years (DALYs) were lost to JEV worldwide in 2005.

### JEV vaccination

Effective JEV vaccination was first developed in 1941 [8] and is now readily available. Laboratory and clinical surveillance data have shown its effectiveness as a public health measure in countries like Japan or South Korea [7]. Data from Thailand and Vietnam show that vaccination has transformed the epidemiology of JEV in those countries, vastly reducing the incidence of JEV since it was introduced or expanded in the 1990s [7], [8]. The vaccine has recently been introduced in Laos but remains to be introduced or rolled out in the routine immunization programs of Cambodia, Laos, and Myanmar. The vaccination of pigs for JEV could be cost-effective from a public health point of view by decreasing the level of circulation in the amplifying host and consecutively the incidence in humans [13], [25]. The impact of mass JEV vaccination of swine on human health has been studied in Japan [26], showing a reduction of JEV transmission to humans. Unfortunately the high turnover of animals and the cost of vaccine make such options cost-ineffective. Implementing swine vaccination in countries like Cambodia or Laos where 70% of all pigs are raised in small-scale farms [27] would be a serious challenge. Although large-scale vaccination of domestic pigs may not be feasible, the use of pigs as sentinel animals appears efficient at least in the early detection of human outbreaks [28].

### Other encephalitides

At least 90 other infectious agents are known to cause encephalitis or severe febrile disease associated with encephalitis signs, several of which circulate widely in Southeast Asia (Table 1).

**Table 1:** Nonexhaustive list of pathogens known to have caused human encephalitis cases published in the literature.

VIRUSES	BACTERIA
**Adenovirus**	*Balamuthia mandrillaris*
Al Khurma virus	***Bartonella henselae, B. quintana*** * or Bartonella bacilliformis*
**Bat lyssavirus**	*Borrelia burgdorferi (Lyme disease)*
California encephalitis	***Brucella*** ** spp.**
Chandipura virus	***Burkholderia pseudomallei***
**Chikungunya virus**	***Chlamydia pneumoniae***
**Cytomegalovirus**	***Chlamydia psittaci***
**Dengue virus**	*Coxiella burneti* (Q Fever)
Eastern Equine Encephalitis virus	***Francisella tularensis***
Enterovirus (incl. EV71)	***Leptospira*** ** spp.**
Epstein-Barr virus	***Listeria monocytogenes***
Hendra virus	***Mycobacterium tuberculosis***
**Herpes simplex virus**	***Mycoplasma pneumoniae***
**Human herpesvirus 6**	***Pasteurella multocida***
**Influenza A virus**	***Streptococcus A pyogenes***
**Influenza A(H5N1) virus**	***Streptococcus*** ** group B**
**Influenza B virus**	*Tropheryma whipplei*
**JC virus**	
Junin virus	**RICKETTSIAE**
Kunjin virus	***Anaplasma phagocytophilum***
Kyasanur Forest virus	***Coxiella burnetii***
LaCrosse Encephalitis virus	***Ehrlichia chaffeensis***
Lassa virus	***Orientia tsutsugamuchi***
Lymphocytic Choriomeningitis Virus	***Rickettsia rickettsii***
Louping Ill virus	***Rickettsia typhi***
**Measles virus**	***Rickettsia felis***
**Mumps virus**	
Murray Valley virus	**PROTOZOA**
**Nipah virus**	Acanthamoeba
**Norovirus**	*Balamuthia mandrillaris*
Omsk hemorrhagic fever virus	*Baylisascaris procyonis*
**Parechovirus**	*Coccidioides immitis*
**Parvovirus B19**	***Cryptococcus neoformans, C. gattii***
**Poliovirus**	***Naegleria fowleri***
Powassan encephalitis virus	***Plasmodia*** ** spp.**
**Rabies and other lyssaviruses**	***Toxocara canis***
**Respiratory Syncytial Virus**	***Toxoplasma gondii***
Rift Valley Fever virus	***Treponema pallidum***
Rocio virus	*Trypanosoma brucei gambiense*
**Rotavirus**	*Trypanosoma brucei rhodesiense*
**Rubella virus**	
Sindbis virus	**HELMINTHS**
St Louis encephalitis virus	*Baylisascaris procyonis*
Tick-borne encephalitis virus	***Gnathostoma*** **spp.**
Toscana virus	***Taenia solium***
Usutu virus	***Trichinella spiralis***
**Varicella Zoster Virus**	***Angiostrongylus cantonensis***
Venezuelan Equine Encephalitis virus	
West Nile virus	**OTHER**
Western Equine Encephalitis virus	**Prion disease**
Yellow Fever virus	

Pathogens ubiquitous or known to circulate in countries of the Mekong region are shown in bold (completed and adapted from Tunkel et al. [91]).

### Dengue encephalitis

The reach of dengue is expanding. The number of symptomatic dengue patients had been estimated at 50–100 million cases each year worldwide [4], [29]. A recent study estimated the global burden of symptomatic cases at 96 million [interval 67.1–135.6] each year, approximately 70% of which occur in Asia and 34% in India alone [30]. This increase from previous WHO estimates is due in part to undernotification [31], [32], but also to increasing urban populations in Southeast Asia [33].

In 2010, WHO reporting schemes recorded 233,912 dengue cases (16,690 deaths) in the Mekong region (excluding southern China) [34], [35]. Dengue was estimated to cause the loss of 420 DALYs per million population per year in Thailand [36]. But a recent study estimated 1,383,120 symptomatic cases [range 513,151–1,805,769] in the Mekong region (excluding China), for an annual burden ranging from 130 DALY per million inhabitants in Vietnam to 1,130 DALY per million inhabitants in Cambodia [37]. This study confirms that dengue is a vast health and economic burden, as found earlier, albeit on a smaller scale [38], [39].

Dengue encephalitis has only recently been accepted as a clinical entity and may manifest itself with signs ranging from fever with limited alteration of consciousness to severe encephalopathy with convulsions or myelitis, often with sequelae in survivors [40]–[46]. Dengue has been associated with encephalitis in 0.5–6.2% of cases. Among 42 Thai patients with dengue encephalitis (DE), three (7.1%) died and one (2.4%) had long-term sequelae [42]. In other DE studies with limited sample sizes (N = 27) in Vietnam, the CFR was 22% and short-term sequelae were observed in 29% of cases but all recovered within seven days [45]. Of nine cases described in another, limited study, also in Vietnam, six (66%) had sequelae at discharge [40].

### Frequent bacterial pathogens and encephalitis

**Bacteria**, including *Mycobacterium tuberculosis* and *Mycoplasma pneumoniae*, tend to be neglected as causes of encephalitis. **Scrub typhus** is an important—yet neglected but treatable—cause of fever in countries of the Mekong region [47], [48]. Of 72 confirmed cases of scrub typhus with clinical records in Songkla, Thailand in the mid-1980s, nine (12.5%) presented with neurological signs. Murine typhus (*Rickettsia typhi*) and leptospirosis (*Leptospira* spp.) may also cause encephalitis [49].

### Rabies encephalitis

**Rabies** is widely enzootic throughout Southeast Asia. Canine rabies is neglected and has re-emerged in China since 1990 [50] (and has even emerged in Flores [51] and Bali [52], [53]), causing high morbidity (as rabid dogs bite more frequently [54]) and economic burden and mortality (human rabies deaths) throughout the region. With an estimated annual incidence of 5.8/100,000 population (95% CI 2.8–11.5) in Cambodia, rabies has been estimated to cause over 800 deaths a year for a population of 14 million [55], an incidence comparable to or higher than that of road-related deaths in developed countries [56]. As with JEV, vaccination is available in the region but generally not readily accessible for populations at risk.

### Rare causes of encephalitis in the Mekong region

Other, less frequent encephalitides circulate in the Mekong region. ***Streptococcus suis*** [57] can cause sporadic and self-limiting outbreaks of meningoencephalitis, even if recent studies in Thailand and Vietnam have shown that the disease may be underestimated [58]. Massive epidemics of encephalitis, such as the enterovirus serotype 71 (**EV71)** encephalitis outbreaks were as well described in Cambodia, Thailand, and Vietnam in 2012 [59]–[61]. Although human cases have not been identified, evidence of **Nipah virus** circulation has been found in bats throughout the lower Mekong Region [62]–[68]. Although it is the—or one of the—main causes of encephalitis in studies conducted in the USA [69]–[71], the United Kingdom [72], France [73] and Australia [74], studies carried out to date in the Mekong Region and data from Cambodia have identified little or no Herpes Simplex Virus (February 2012 conversations with P. Buchy, personal communication). Although this relatively lower incidence may perhaps be overshadowed by the larger incidence of tropical encephalitides, diagnosing this pathogen remains a priority as it is one of the few accessible to curative management, despite its incomplete effectiveness even in developed countries [75]–[77].

### Estimating the financial burden of encephalitis

The **financial** burden of encephalitis is significant: post-exposure rabies vaccination and travel costs to access it can amount to a Cambodian farmer's monthly wage (A. Tarantola, unpublished data), while the direct and indirect costs of JEV encephalitis hospitalization and care can amount to nearly ten times that [22]. Hospitalization for dengue costs half a month's average wage in rural Cambodia [39]. Encephalitis survivors often present severe sequelae; consequences on a family's income, organization, or balance can be dire.

### The impact of encephalitides in domestic animals

Encephalitic zoonoses impact animal husbandry as well. Japanese encephalitis in pigs causes stillbirths, abortions, infertility, and small litter sizes [78]. Furthermore, in Thailand for example, veterinary authorities are obliged by law to cull pigs after a JEV or a *S. suis* outbreak, causing further significant financial losses to pig producers [79]. The circulation of Japanese encephalitis in pigs in the Region is intense, principally in the countryside but increasingly in urban areas as well [80], [81]. Outbreaks of Nipah virus would cause fatal pneumonia in pigs and would result of culling thousands of pigs in countries with veterinary law enforcement (like Thailand or Vietnam) as it happened in Malaysia in 1998 [68]. To the best of our knowledge, the economic impact of these diseases in animals and their prevention has not been evaluated in the Mekong region.

### Perspectives

Over the past six decades, research has barely improved our understanding of enzootic circulation, vector control and etiological curative measures for JEV [82]. It is very likely that expansion of JEV vaccination to the remaining JEV endemic areas would be a highly cost-effective intervention [22]. In the absence of vaccination, the encephalitis burden will likely increase in the coming years in most Southeast Asian countries, especially as semi-commercial systems of pig production develop in periurban areas [83] with growing populations [12] and increasing land use for agriculture [84], [85].

Whatever the nature and scope of the encephalitis burden in Southeast Asia and the criterion used to assess it—morbidity, mortality, handicap, impact on families' budget, or livelihood—that burden is borne principally by the same rural, economically fragile populations with limited access to prevention and health care. The identification of etiological agents is useful for estimating burden and to guide research, but first and foremost to urgently rule out treatable or preventable causes. Overall, surveillance and diagnostic capabilities for encephalitis remain poor in most areas of the Mekong region and beyond [6]. Although the burden of non-infectious, autoimmune encephalitis [86] in the region remains to be ascertained, the best laboratories worldwide continue to only identify etiological infective agents in around 50% of cases at most [72], [73], [82], [87]–[89]. Little progress in bedside diagnostic capacities is to be expected to help treat patients in frontline care.

Probabilistic encephalitis management protocol based on sound clinical, epidemiological, and laboratory-based research would benefit patients in settings with limited access to diagnostic capacities. State-of-the-art medical and scientific collaborations dedicated to encephalitides in the Mekong region are urgently needed. These may be organized around three main axes: (i) reinforced microbiological and immunological etiological investigations, (ii) improved clinical and therapeutic management, and (iii) assessment of ecoepidemiological and social determinants to identify environmental and behavioural risk factors for acquiring disease [90]. Well-designed frontline diagnostic strategies using modern biomolecular tools with high sensitivity complemented by highly specific testing by second- or third-line, highly competent research laboratories would provide timely diagnosis of immediately treatable aetiologies, to the patients' direct benefit. Microbiological and biomolecular approaches would help describe the distribution of pathogens and open possibilities for improved diagnosis and treatment. Encephalitis, however, is about individuals, families, and communities. Epidemiologists would make the irreplaceable laboratory findings more accurate, representative, and interpretable through rigorous selection and documentation of patients. They would also add further value to these findings by helping identify hotspots, investigate trends and outbreaks, assess sequelae or associated risk factors, and generate a database and map documenting which pathogen was identified where. Finally, ecoepidemiological investigations of what are often zoonotic pathogens using a one-health approach would provide insights on pathogens' circulation and prevention on a larger scale.

Such studies and collaborative projects are urgently needed to improve human capacity, diagnostic techniques, and strategies, and to further document the macroepidemiology of encephalitis as well as the microepidemiology of certain pathogens, whether known or yet to be discovered, in the Mekong region.

**Key Learning Points**

- Even in the best laboratories, aetiological agents are diagnosed in only about 50% of patients with encephalitis in Asia.
- Japanese encephalitis virus accounts for 50% of the diagnosed pathogens in areas where vaccination is not universally accessible.
- The burden of disease is particularly borne by children of rural, economically fragile families.
- Multidisciplinary research may help improve knowledge and practice in detecting, treating, and preventing encephalitis in the Mekong region.

**Five Key Papers in the Field**

- Mackenzie JS (2005) Emerging zoonotic encephalitis viruses: lessons from Southeast Asia and Oceania. J Neurovirol 11: 434–440.
- Erlanger TE, Weiss S, Keiser J, Utzinger J, Wiedenmayer K (2009) Past, present, and future of Japanese encephalitis. Emerg Infect Dis 15: 1–7.
- Solomon T, Dung NM, Vaughn DW, Kneen R, Thao LT, et al. (2000) Neurological manifestations of dengue infection. Lancet 355: 1053–1059.
- Granerod J, Ambrose HE, Davies NW, Clewley JP, Walsh AL, et al. (2010) Causes of encephalitis and differences in their clinical presentations in England: a multicentre, population-based prospective study. Lancet Infect Dis 10: 835–844.
- Coker RJ, Hunter BM, Rudge JW, Liverani M, Hanvoravongchai P (2011) Emerging infectious diseases in southeast Asia: regional challenges to control. Lancet 377: 599–609.
